# Future use-cases of vaccines in malaria control and elimination

**DOI:** 10.1016/j.parepi.2020.e00145

**Published:** 2020-05-06

**Authors:** Melissa A. Penny, Flavia Camponovo, Nakul Chitnis, Thomas A. Smith, Marcel Tanner

**Affiliations:** Swiss Tropical and Public Health Institute, Basel, Switzerland; University of Basel, Basel, Switzerland

**Keywords:** *Malaria*, *Plasmodium falciparum*, Vaccines, Use-cases, Innovation

## Abstract

Malaria burden has significantly changed or decreased over the last 20 years, however, it remains an important health problem requiring the rigorous application of existing tools and approaches, as well as the development and use of new interventions. A malaria vaccine has long been considered a possible new intervention to aid malaria burden reduction. However, after decades of development, only one vaccine to protect children has completed phase 3 studies. Before being widely recommended for use, it must further demonstrate safety, impact and feasibility in ongoing pilot implementation studies. Now is an appropriate time to consider the use-cases and health targets of future malaria vaccines. These must be considered in the context of likely innovations in other malaria tools such as vector control, as well as the significant knowledge gaps on the appropriate target antigens, and the immunology of vaccine-induced protection. Here we discuss the history of malaria vaccines and suggest some future use-cases for future malaria vaccines that will support achieving malaria health goals in different settings.

## 1 Malaria control and elimination

In 2015, the global technical strategy (GTS) of WHO called for elimination of malaria in 20 countries by 2025, and 35 by 2035, and reduction of at least 90% of clinical incidence and mortality in the remainder (Bhatt et al., 2015). These targets were set following dramatic reduction over 15 years of malaria incidence, mainly as a result of integrated large scale-up of insecticide treated nets, indoor residual spraying, and artemisinin combination therapy (Bhatt et al., 2015). In many areas where previously most people were infected for most of the time, prevalence has become patchy, and the burden of disease has shifted from the youngest children to older age-groups (Mogeni et al., 2016; Nkumama et al., 2017) .

Recently, however, overall progress in reducing *Plasmodium falciparum* mortality and clinical incidence has stalled (World Health Organization, 2017) and the WHO world malaria report estimated that 2016 there was an increase in clinical cases in many regions. History shows that there are many potential causes of resurgence (Cohen et al., 2012), including resistance, reductions in intervention coverage reflecting funding gaps, and also the intrinsic dynamics induced by many interventions (such as delaying acquisition of natural immunity), which have maximal impact soon after scale-up.

Drug resistance is currently a particular worry in the Mekong, with artemisinin resistance and resistance to partner drugs present and increasing (Ménard et al., 2016). There is concern about artemisinin resistance spreading to, or emerging in, Africa, as was seen with chloroquine in the 1990s. In addition, insecticide resistance is becoming a major concern across Africa (Minetti et al., 2020; Moyes et al., 2019).

Novel insecticide and/or vector controls, as well as new drugs, may soon enter phase 3 studies, however, the development pathway for these is potentially long and uncertain, making the timeline to availability, as well as the affordability of these tools, unsure. Other new tools such as monoclonal antibodies are also in very early development, and their future product development pathway or potential protection will become apparent with forthcoming early clinical studies (Kisalu et al., 2018; Tan et al., 2018).

## 2 Expectations and realities of malaria vaccines to date

A malaria vaccine has long been seen as a potential game changer in the fight against malaria, although vaccine development began at a time when the emphasis in international health was shifting from immediate goal of world-wide malaria eradication to control, with the Alma-Ata agreement focusing on primary health care (World Health Organization, 1978). In the shadow of the war in Vietnam, the main institutional support for a malaria vaccine in the 1970s came from the US military's Walter Reed Army Institute of Research, with the hope being that a very short course of vaccination could provide complete protection against infection for the military and short-term visitors to endemic areas. Following on the pioneering work of the Nussenzweigs (Nussenzweig and Nussenzweig, 1984; Nussenzweig et al., 1967), the early candidate vaccines were based on sporozoite antigens, however it became evident that it was not straightforward completely blocking infection with such a vaccine (Ballou et al., 1987; Hoffman et al., 1987). It was not until 1997 that partial efficacy of the main candidate, RTS,S (based on the *P. falciparum* circumsporozoite surface protein, and known as a pre-erythrocytic or anti-infective vaccine), could be established in humans (Stoute et al., 1997).

There was a perception that vaccines targeting other antigens, especially those of the blood stages of the parasite, might be more successful (Saul et al., 1999; Genton and Reed, 2007). These were perceived as protecting the most vulnerable from disease, children under 5 years of age in endemic areas, who suffered the bulk of severe malaria and mortality. A leaky vaccine appeared an option, especially if responses to blood stage antigens could be used to reduce the incidence of severe disease, even if transmission was barely affected. But during the 1990s, few vaccine candidates reached the field; mainly phases 1 and 2a, b and only one, SPf66, undertook a phase 3 trial in infants (Genton and Reed, 2007; Acosta et al., 1999a). The latter trial of SPf66 supported learnings and thus guidance on how to carry out GCP and DSMB clinical studies on infant vaccines in a rolling design from phase 1 to 3 including investigating safety and immunogenicity with EPI vaccine interactions (Acosta et al., 1999b). Those vaccine candidates based on synthetic peptides (Alonso et al., 1994; Nosten et al., 1996) or recombinant proteins that combined sporozoite antigens with blood stages (Saul et al., 1999; Stuerchler et al., 1995; Genton et al., 2002), had mixed results. The availability of the malaria genome opened up the prospect of DNA vaccines (Doolan et al., 1997) encoding a wide range of malaria antigens, but initial clinical studies of these were no more encouraging (Epstein et al., 2002; Long and Hoffman, 2002) and prime-boost strategies with adenoviral vectors and modified vaccinia virus (Dunachie and Hill, 2003; Bejon et al., 2005) were also disappointing.

When substantial funding again became available for malaria vaccine development at the start of this century, RTS,S remained the most advanced candidate. With the realisation that preventing initial infection of the human could be an effective approach in reducing disease burden it was reconceptualised as a morbidity control tool for young children, and enrolled in a clinical development pathway aiming at licensure as a paediatric vaccine. Delivery of RTS,S to infants or children via the expanded program of immunization (EPI) also offered a novel strategy for delivering malaria interventions, and is a proven mechanism for reaching children with high coverage of DTP3 vaccines in many countries that are also endemic with malaria. RTS,S consistently demonstrated partial protection in a series of randomized controlled trials (Bojang et al., 2001; Alonso et al., 2004; Bojang et al., 2009; Agnandji et al., 2011; Agnandji et al., 2012; RTSS Clinical Trials Partnership, 2015; Abdulla et al., 2008), remaining ahead of a growing field of other potential vaccines. Now administered with the AS01 adjuvant, RTS,S completed phase 3 clinical studies in 2015, culminating in the largest clinical studies in children ever conducted in Africa (RTSS Clinical Trials Partnership, 2015).

The long delays inherent in vaccine development have always meant that we are testing vaccines that are aligned with priorities set at least a decade previously. When the goal of eradication returned to the fore in the last decade (Roberts and Enserink, 2007), achieving sterile protection again became a focus of vaccine developers (Hoffman et al., 2015). However the phase 3 study of RTS,S/AS01 in over 16,000 children in seven African countries demonstrated only moderate clinical efficacy over the 32 months of follow-up with a higher efficacy in a 5–17 month-old cohort compared to a 6–12 week cohort (RTSS Clinical Trials Partnership, 2015). Extensive modelling results indicated that routine immunization of children with RTS,S/AS01, is potentially cost effective in areas of moderate to high prevalence (Penny et al., 2016) depending on other interventions in place, and RTS,S/AS01 received a positive scientific opinion from the European Medicines Agency in 2015. However the protection profile of RTS,S/AS01 is not satisfactory, with rapid waning of antibody levels and efficacy declining over time (RTSS Clinical Trials Partnership, 2015; Penny et al., 2015; White et al., 2015). The World Health Organization recommended further pilot studies to evaluate safety, impact, and feasibility in implementation (World Health Organization, 2016), and these began in Ghana, Kenya, and Mali at in the first half of 2018, aiming to vaccinate roughly 360,000 children per year via routine systems (World Health Organization, n.d.; https://www.who.int/malaria/media/malaria-vaccine-implementation-qa/en/, n.d.). These pilot studies will estimate impact on severe disease and potentially mortality in real implementation outside highly controlled clinical studies and provide insights into implementation of a malaria vaccine at large. This means we will gain estimates of vaccine effectiveness, not just clinical efficacy as observed in the Phase 3 studies. The policy recommendation and the future position and role of RTS,S/AS01 for childhood vaccination will depend on these pilot studies.

The last decade has consequently seen a recalibration of what can be expected from vaccines (malERA Refresh Consultative Panel on Tools for Malaria Elimination, 2017; The malERA Consultative Group on Vaccines, 2011) and in the context of the GTS elimination targets, morbidity control in the youngest children may no longer be the most promising use case for a partially protective pre-erythrocytic vaccine like RTS,S.

## 3 Innovation and the future

The vaccine candidates currently in development (Draper et al., 2018) include anti-infective vaccines other than RTS,S, such as attenuated or irradiated whole sporozoite vaccines (PfSPZ), or RTS,S like vaccines such as R21 (Collins et al., 2017). Potentially potent monoclonal antibodies identified are generally also anti-infective antibodies as they come out of decades of anti-infective vaccine development.

There remains a need for the identification of new targets and chemistries for malaria vaccines, but until now the health returns on investments in vaccine development and clinical studies have been less than satisfying. Nevertheless, they have delivered enormous knowledge gains and impressive technical advances, in the fields of both cellular and humoral immunology (Draper et al., 2018; Beeson et al., 2019), and recently it is has been recognized there is pertinent need to focus on obtaining more understandings of malaria immunity, protection and relationship with pre-exposure (Beeson et al., 2019). Controlled human malaria infection studies have driven advances in both systems biology and immunology that are valuable not just for malaria vaccine and anti-malaria drugs, but also for other diseases.

Many bio-medical research questions remain to be answered. The search continues for bio-markers or immune correlates of protection that can be easily measured substantially reducing the costs of clinical development. The challenge that this represents is highlighted by recent studies on the variation in immune responses between populations (Kurtovic et al., 2019) and findings that the clinical outcomes of vaccination may be largely driven by individual-level variation in immune responses. Innate host immune fitness may be of critical importance, both in determining the response to vaccination, and vulnerability to disease.

Rather than focusing on only identifying and developing new antigens, efforts should also center on understanding the limitations and successes of vaccines been done by re-analyzing immunological samples from past studies to understand the roles of individual and population genetics or immune fitness. For example, a recent study undertook a comprehensive reanalysis of blood samples from Tanzanian and Mozambican children in the RTS,S malaria vaccine phase 3 trial. Immune profiling and transcriptomic analysis identified factors, including age, location and anemia, that shape immune development, and further compared results to Dutch children (Hill et al., 2020). Such important studies require public sharing of samples and data, analogously to renewed analysis of old drugs in the area of chemotherapy.

Prioritisation of new products should be driven by rational criteria, and vaccines with short durations of effect, requiring a high number of doses, are less likely to meet criteria of cost-effectiveness or budget impact analyses. The economics of manufacturing, including both production costs and economies of scale should not be ignored. But innovation is not only taking place in basic science and product development, but also in its translation to endemic countries. Clinical studies in Africa have led to huge advances in the technical capacity of local laboratories. Even the Good laboratory practices mosquitoes required to produce PfSPZ irradiated sporozoites are being produced in endemic countries. Innovations are making novel delivery mechanisms practicable, such as skin patches, which could reduce the numbers of contacts required with health staff during the vaccination schedule. For this reason, a novel product that currently appears to be operationally infeasible should not be a priori precluded from testing and development. Innovation in the use of mathematical modelling will also support exploring multiple use cases and to iteratively support assessing candidates, this includes going beyond traditional scenario analysis and using modelling early in development rather than when vaccines are already in clinical studies.

**Fig. 1:**
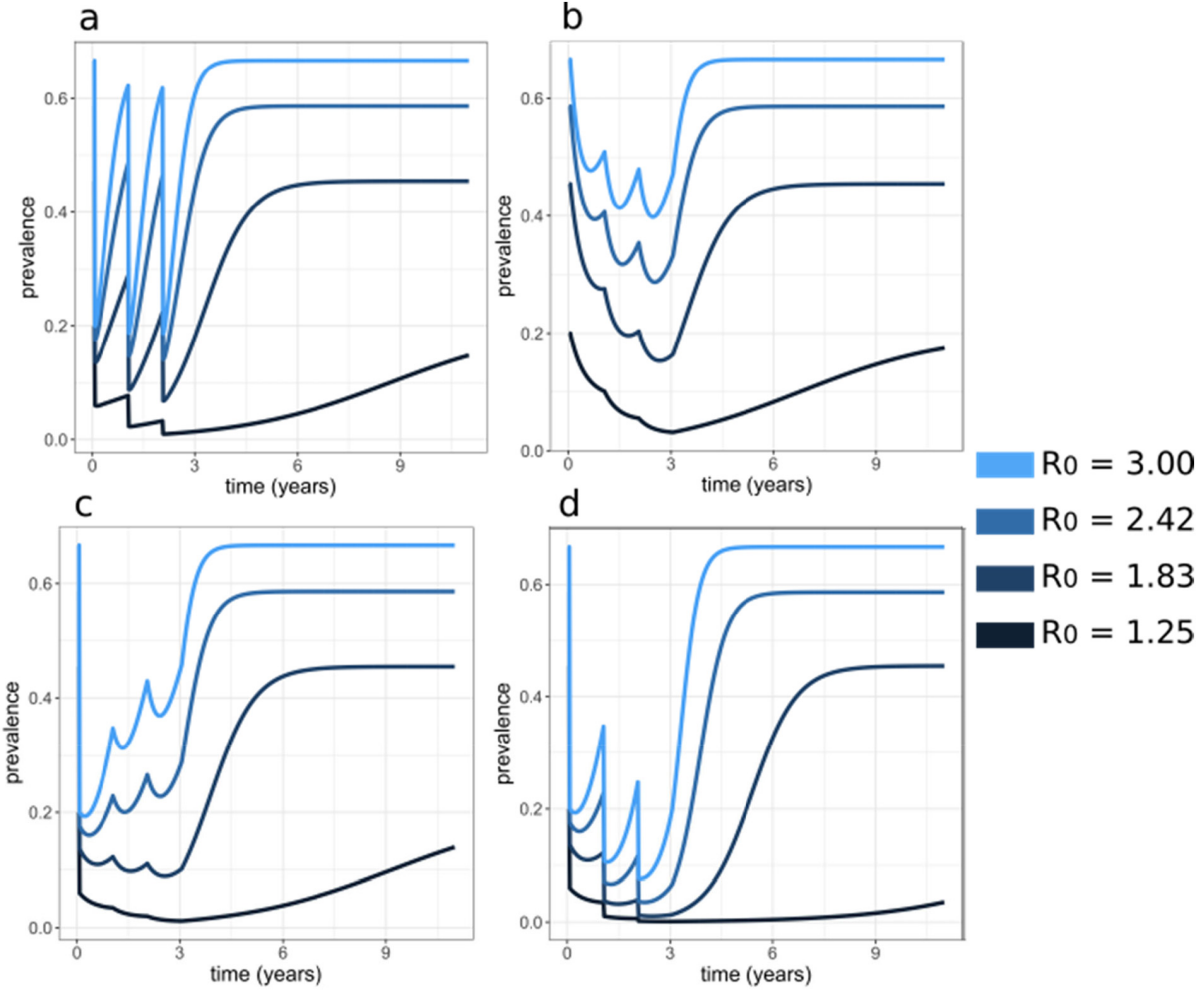
Predicted all age prevalence during and following three years of mass interventions for different strategies a) MDA alone for three years (one dose per year), b) vaccine alone for three years, c) vaccine for three years with drugs only the first year, d) vaccine and drugs every year for three years. Coverage of 70% for each intervention was assumed over a range of initial ***R***_**0**_ values from 1 to 3. Initial ***R***_**0**_ is indicated by colour, with the darker the colour the lower the transmission level.

**Fig. 2:**
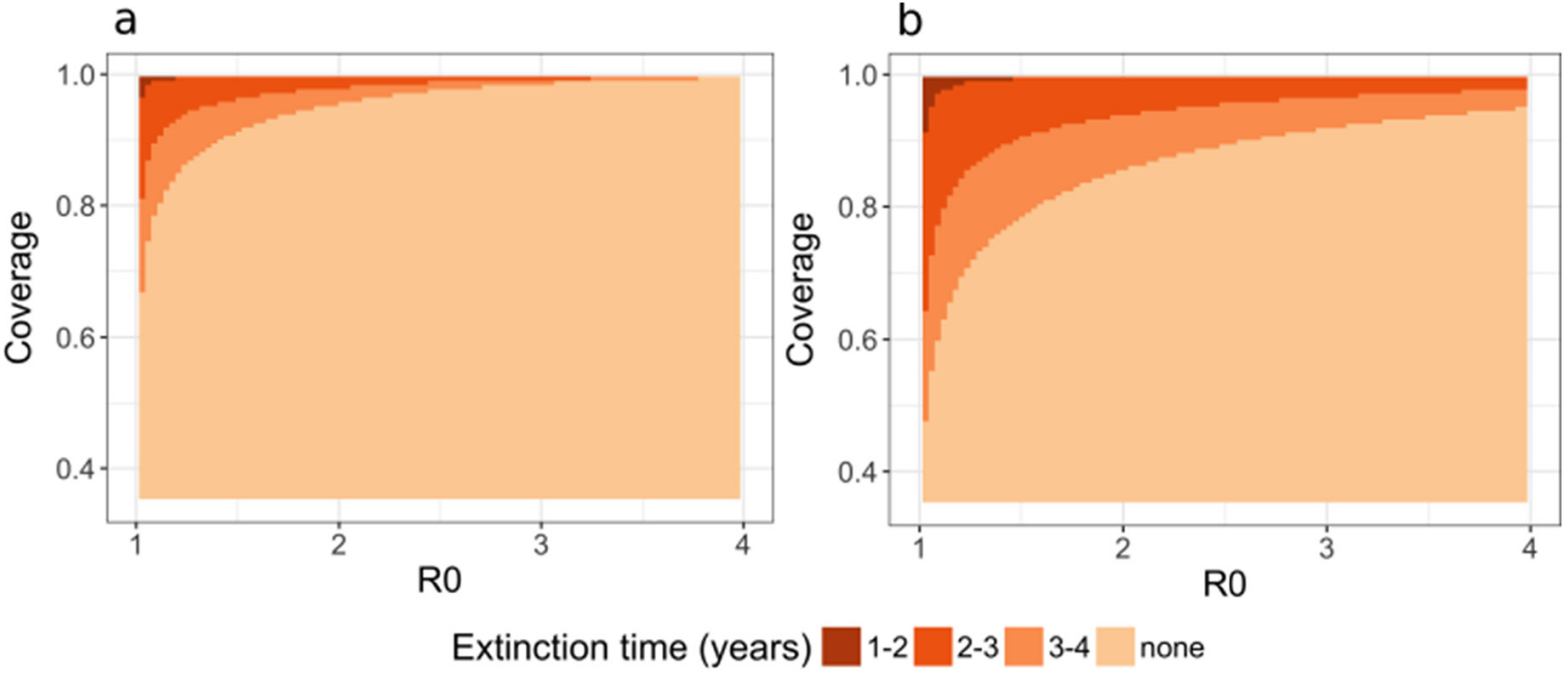
Visualisation via heat map of the estimated time until transmission is interrupted (prevalence below 0.1%) for a range of coverages and transmission levels as a result of three years of mass interventions for a) MDA alone for three years or b) mass vaccination with MDA for each year, up to three years. Estimates of malaria interruption time for a range of yearly intervention coverage and initial ***R***_**0**_, with colour indicating time to interruption with the darker the colour indicating earlier interruption. No interruption occurred after 4 years as interventions ceased by year 3. No interruption of transmission was observed in the vaccination only strategies.

All this requires innovation in firstly identifying priority public health needs and secondly defining the use-cases for vaccines or monoclonal antibodies to achieve these health goals, thus identifying the settings, target ages-groups or populations, and the system of delivery to the population. These use-cases require consideration of health outcomes and needs other than incidence of clinical malaria in children and lives-saved or mortality (Moorthy et al., 2013), for instance the objectives might be to reduce the time to elimination, or delay resurgence (Box 1, Fig. 1, Fig. 2). In turn, this means that people of all ages might need to be vaccinated. The next sections of this paper outline the state of knowledge for some of these alternative future use cases.

Box 1 **Estimated impact of mass vaccination with and without mass drug administration.**

Using a modified Ross-Macdonald model of malaria (Dietz, 1988; Allen, 1994) the effects of mass vaccination alone were compared to mass drug administration alone, or to the combination of both interventions.. The model is represented by a set of difference equations describing susceptible-infected-susceptible malaria dynamics with the number of infectious individual, *I*_*t*_, at time *t*, in the absence of an intervention represented by

(1) $$I_{t+1}=\frac{\beta }{N}I_{t}\left(N-I_{t}\right)+\gamma I_{t}$$

Here *β* (*β* > 0) is the transmission parameter representing the number of new infections per infected individual at the next time step, *γ*(0 < *γ* < 1) is the proportion of population that remains infectious at the next time, and *N* the total human population. Case-management is incorporated by decreasing *γ* and thus accounting for removal of infections through standard case management. We assume the length of infection and thus infectious period of 200 days. The susceptible population is given by *S*_*t*_ = *N* − *I*_*t*_. The basic reproduction number a measure of initial transmission intensity in this model, represents the number of secondary infections resulting from a single infection, and in this simplified model with no intervention is given by

(2) $$R_{0}=\frac{\beta }{1-\gamma }.$$

We model mass treatment and mass vaccination with a anti-infective vaccine by altering (1) to include terms representing infection prevention and parasite clearance, namely,

(1) $$I_{t+1}=\left(1-\epsilon_{t}\right)\frac{\beta }{N}I_{t}\left(N-I_{t}\right)+\left(1-\rho_{t}\right)\gamma I_{t}$$

where *ρ*_*t*_ represents probability of clearing infections in the infectious population and is a combination of MDA coverage and decay of drug efficacy, such that with no MDA *ρ*_*t*_=0, and if coverage and efficacy were 100% then *ρ*_*t*_=1. The term *ϵ*_*t*_ represents the probability of preventing new infections in the susceptible population and is given by vaccination coverage and probability of prevention against infection at time *t* when the strategy includes vaccination, or is given by drug coverage and short-lived efficacy of prophylaxis if drugs are delivered without vaccination.

Three yearly applications of one round of each strategy were deployed in the models, with strategies including vaccination assuming 3 doses in the first year of vaccination (as is with current childhood vaccination in the Pilot studies or SMC vaccine trials), to reach a high initial efficacy (85–91%) against infection with RTS,S or similar vaccine (Penny et al., 2015; White et al., 2015), Vaccine efficacy was assumed to decay bi-phasically such that initial decay is quick followed by a slower decay, with a half-life of approximately 7 months (Penny et al., 2015). Drug efficacy of 100% was assumed with immediate clearance of blood stage parasites with an additional prophylaxis effect preventing infection that decayed exponentially with a half-life of 10 days. When drugs were deployed with vaccines, drugs were given either for all three years, or only the first year.

We simulated impact of the interventions on the number of infectious individuals and thus calculated all age prevalence as well as time till interruption of transmission (if it occurred). We assumed perennial transmission, a population of 10,000 individuals, and simulate a range of *R*_0_ (by altering *β*) and a range coverages of 3 years of mass intervention strategies. Disease dynamics were followed for 10 years and interruption of transmission was assumed to occur if prevalence was below 0.1%. Mosquito dynamics were not modelled explicitly as these vaccines and drug based interventions are assumed to not kill mosquitos and thus do not alter the force of infection *β*. Stochasticity can be added to the model, but will have little effect on the overall conclusions, and only adds variation to estimates of extinction time. This simple model account for vaccine effect and decay, but does not account for natural immunity development and decay, nor the vast range of heterogeneities expected in malaria such as age, transmission, spatial effects etc. However, the model is sufficient as a cornerstone to begin to explore dynamics of combining interventions and prompt analysis with more detailed models.

The results suggest that if interruption of transmission is not achieved prevalence resurgences to initial levels (Fig. 1), consistent with previous modelling results for MDA (Brady et al., 2017). At 70% coverage, a lower level of prevalence is achieved in interventions including drugs, indicating mass vaccination is less effective than MDA alone in overall reduction of transmission. Adding mass vaccination to MDA strategies will potentially 1) result in a lower level of prevalence at the end of 3 years (compare Fig. 1b and c–d), 2) keep prevalence levels lower for longer (compare Fig. 1a and d), 3) result in being more likely to interrupt transmission for lower coverages (Fig. 2), and 4) possibly achieve interruption in shorter time periods (Fig. 2). Using drugs with vaccination in all three rounds is better than for only one round (Fig. 1c–d). We conclude there is added, and possible synergistic benefit to consider dual rollout of MDA and anti-infective vaccines.

Alt-text: Box 1

## 4 Alternative use cases

### 4.1 All-age vaccination to support local elimination programs

The immediate future of malaria vaccines will be to move beyond the childhood age group on which clinical development has so far focused. Mathematical models of generic anti-infective vaccines have indicated that these have the potential to dramatically reduce transmission when used in as a mass intervention in low prevalence settings (Penny et al., 2008). If the initial prevalence is very low, vaccination might interrupt transmission in isolated communities (Penny et al., 2008). In this vein, a trial of mass vaccination with PfSPZ has been planned in Bioko Island (Olotu et al., 2018), Equatorial Guinea aiming to test its role in an intervention package for elimination. Now that we know the profile of the effect of RTS,S, it is possible to estimate likely impacts in specific places to reduce burden, and where inclusion of mass vaccination in surveillance response packages may effectively drive elimination.

It will be important to balance operational feasibility, in terms of delivery of the required number of doses in mass campaigns, with impact and cost-effectiveness. Additional hurdles in using any vaccine in mass campaigns are questions about immune responses, longevity and protection in adults, across populations and settings, and in vulnerable populations such as pregnant women, malnourished individuals and/or HIV-infected individuals, as well as compliance which may be lower than for drug administration campaigns. Therefore, more evidence for the impact and cost effectiveness of these anti-infective vaccines when used in combination with drugs and reactive interventions in aiding elimination must be collected, but the potential use of a vaccine under these intervention strategies must be considered seriously in light of the current complex situation for malaria.

### 4.2 Combination with or alternative to seasonal malaria chemoprevention for morbidity and mortality prevention

There has been recognition of the potential impact of adding/integrating anti-infective vaccines like RTS,S to existing intervention strategies (Greenwood et al., 2017). Seasonal malaria chemoprevention (SMC) is used to protect children from 0–5 (and recently 0–10), with the idea being to treat these children in the seasonal treatment of malaria with 1–3 rounds of a drug which clears and then prevents infection. Despite protection being shorter lived than expected, RTS,S offers longer term prophylaxis than any existing drugs, and it has been recognized as a possible alternative to or in addition to drugs in seasonal malaria chemoprevention (SMC) (Greenwood et al., 2017). Different combinations are currently being trialed in Mali and Burkina Faso, with questions concerning whether a vaccine can replace SMC treatment or increase impact if added in addition. It is not obvious how the effectiveness of such a strategy compares with SMC drugs alone, whether it is synergistic with the drug treatments, whether the addition of a vaccine can reduce the number of visits through a child's early years, and whether the cost effectiveness of these strategies compare. In addition, since strategies like SMC only address seasonal malaria settings, there is a need to develop solutions for non-seasonal settings. This will require longer-acting high efficacy vaccines or monoclonal antibodies (with or without drugs).

### 4.3 Combination with mass drug administration for elimination and preventing infection

The use of vaccines as mass interventions, in addition to and combined with drug administration but not as a substitute, may make otherwise unachievable elimination targets reachable, especially when combined with surveillance response packages. Recently this has been considered for aiding elimination in artemisin resistance areas such as the Mekong (Thu et al., 2017), and vaccination campaigns in addition to MDA might be considered (Gosling and von Seidlein, 2016).

Box 1 below uses a simple model of malaria dynamics to illustrate the likely effects of the use of an anti-infective vaccine in combination with mass-drug administration (MDA). Fig. 1, Fig. 2 suggest there is potential of synergism between the two interventions, with the addition of a vaccine increasing public health impact and chances of elimination. The mass drug action component clears a large number of infections from a population and vaccine, (even with duration of protection against infection less than one year) has a substantial impact on delaying resurgence. Further investigations are needed with models accounting for complex heterogeneities in immunity, malaria transmission, including importation of infections, seasonality and different health system strengths. But our preliminary results prompt questions to be asked about the combination tool use cases of anti-infective vaccines.

### 4.4 Combination of different vaccine effects for several health goals

A polyvalent vaccine directed at several stages of the parasite lifecycle, has long been seen as a potentially transformative intervention (Parker et al., 2001). In principle, anti-infective effects, blocking of merozoites or trophozoites, and/or and effects against gametocytes within humans, or blocking sporozoite development within the mosquito, might be combined in the same product. But this remains a distant goal that should be pursued only with the awareness that the limits to vaccine impact imposed by focusing on a single stage may be less important than the duration of effect and the costs and feasibility of deployment.

Over the last 5–10 years there has been an increase in pre-clinical research on vaccines using gametocyte, or mosquito-stage antigens (Draper et al., 2018). However, unless these have a long duration effect, a transmission-blocking vaccine would have limited value as a stand-alone intervention. Short-term transmission-blocking can already be achieved using ivermectin (which kills mosquitoes (Chaccour and Rabinovich, 2019)), or chemotherapy (which also clears infections). Co-administration of ivermectin with dihydroartemisinin-piperaquin e (Smit et al., 2018) thus achieves much of what a transmission blocking vaccine could do. Monoclonal antibodies which have much longer duration of effect than drugs, may also become an alternative.

Such vaccines raise specific, but surmountable issues for regulatory agencies because they have no direct individual benefit (Healer et al., 2017) and it is likely that they would be combined with another agent such as a sporozoite vaccine. Modelling suggests that this combination will have more impact and may be more cost-effective than anti-infective vaccines alone, especially for mass vaccination in low transmission settings (Penny et al., 2008; Tediosi et al., 2009). Both a pre-clinical and modelling studies have suggested that there are likely to be synergistic effects of this vaccine combination (Sherrard-Smith et al., 2018). An additional value of combination vaccines is that the transmission-blocking component may protect against evolution of vaccine insensitivity, by blocking onward transmission of any parasites insensitive to anti-infective component (Smith et al., 2011; Smith et al., 2008).

### 4.5 Targeting vulnerable populations and outbreaks to prevent transmission

In the longer term, for settings where burden continues to decline and elimination targets are met, anti-infective vaccines maybe an appropriate intervention for protecting specific vulnerable groups such as pregnant women, travellers, non-immune populations exposed to outbreak risks, or populations in conflict zones where healthcare systems and surveillance response have broken down. Further applications may also include prevention of resurgence or prevention of reintroduction of malaria in areas recently eliminated or close to elimination, especially if other interventions such as vector control are no longer in use. This use-case, in particular for conflict areas or containing outbreaks, will necessarily define simple administration best with single doses of vaccines or monoclonal antibodies.

## 5 Concluding remarks

In 2014, the WHO set the preferred characteristics of malaria vaccines: with an efficacy of 85% and duration of protection of a least two years (World Health Organisation, 2015). Since this time, malaria burden, research and development of new tools and our understanding of malaria vaccines and immune responses have significantly changed. The needs for malaria and future tools, use-cases and key characteristics of vaccines should be revisited. The most important discussion moving forward will not necessarily be to set minimum efficacy or duration targets for vaccines to reach, but rather to set the health goals malaria vaccines will support and to define use-cases to achieve these that combine vaccines with other interventions, be they vector control, chemoprevention or chemotherapeutic, that support achieving our malaria health goals in different settings. Key determinants of the effectiveness and thus impact of vaccines and any new malaria tool will be the feasibility of deployment, coverage, compliance and adherence, and should be incorporated into the use cases and product specifications. Only then should the preferred characteristics of vaccines with regard to efficacy and duration of effect be considered.

## Funding

MAP and FC were funded by the 10.13039/501100001711Swiss National Science Foundation through the SNSF Professorship of MAP (PP00P3_170702). TAS and NC received support from 10.13039/100000865Bill and Melinda Gates FoundationOPP1032350.

## Authors' contributions

MAP drafted the manuscript. MAP, TAS and MT wrote the final manuscript. All authors edited and approved the final version. MAP carried out the modelling and analysis.

## Declaration of competing interest

MAP was funded by her Swiss National Science Foundation Professorship PP00P3 170702. MAP declares funding from PATH's Malaria Vaccine Initiative to support modelling for the Malaria Vaccine Implementation Program and past funding from the Bill and Melinda Gates Foundation. TAS declares funding from Bill and Melinda Gates OPP 1032350. MT declares past funding from the Bill and Melinda Gates Foundation and from PATH-MVI; the latter in the frame of the RTS,S clinical development. All other authors declare that they have no competing interests.
